# Genome-Wide Association Study in Asian Populations Identifies Variants in *ETS1* and *WDFY4* Associated with Systemic Lupus Erythematosus

**DOI:** 10.1371/journal.pgen.1000841

**Published:** 2010-02-12

**Authors:** Wanling Yang, Nan Shen, Dong-Qing Ye, Qiji Liu, Yan Zhang, Xiao-Xia Qian, Nattiya Hirankarn, Dingge Ying, Hai-Feng Pan, Chi Chiu Mok, Tak Mao Chan, Raymond Woon Sing Wong, Ka Wing Lee, Mo Yin Mok, Sik Nin Wong, Alexander Moon Ho Leung, Xiang-Pei Li, Yingyos Avihingsanon, Chun-Ming Wong, Tsz Leung Lee, Marco Hok Kung Ho, Pamela Pui Wah Lee, Yuk Kwan Chang, Philip H. Li, Ruo-Jie Li, Lu Zhang, Wilfred Hing Sang Wong, Irene Oi Lin Ng, Chak Sing Lau, Pak Chung Sham, Yu Lung Lau, Asian Lupus Genetics Consortium (ALGC)

**Affiliations:** 1 Department of Paediatrics and Adolescent Medicine, LKS Faculty of Medicine, The University of Hong Kong, Pokfulam, Hong Kong; 2 Joint Molecular Rheumatology Laboratory of the Institute of Health Sciences and Shanghai Renji Hospital, Shanghai Institutes for Biological Sciences, Chinese Academy of Sciences, and Shanghai Jiaotong University School of Medicine, Shanghai, China; 3 Department of Epidemiology and Biostatistics, Anhui Medical University School of Public Health, Hefei, Anhui, China; 4 Department of Medical Genetics and Key Laboratory for Experimental Teratology of the Ministry of Education, Shandong University School of Medicine, Jinan, Shandong, China; 5 Lupus Research Unit, Department of Microbiology, Faculty of Medicine, Chulalongkorn University, Bangkok, Thailand; 6 Department of Medicine, Tuen Mun Hospital, New Territory, Hong Kong; 7 Department of Medicine, LKS Faculty of Medicine, The University of Hong Kong, Pokfulam, Hong Kong; 8 Department of Medicine, Pamela Youde Nethersole Eastern Hospital, Chai Wan, Hong Kong; 9 Department of Paediatrics and Adolescent Medicine, Tuen Mun Hospital, New Territory, Hong Kong; 10 Department of Medicine, Queen Elizabeth Hospital, Kowloon, Hong Kong; 11 Department of Rheumatology, Anhui Provincial Hospital, Hefei, Anhui, China; 12 Department of Medicine, Faculty of Medicine, Chulalongkorn University, Bangkok, Thailand; 13 Department of Pathology, LKS Faculty of Medicine, The University of Hong Kong, Pokfulam, Hong Kong; 14 Department of Psychiatry, LKS Faculty of Medicine, The University of Hong Kong, Pokfulam, Hong Kong; The Wellcome Trust Centre for Human Genetics, University of Oxford, United Kingdom

## Abstract

Systemic lupus erythematosus is a complex and potentially fatal autoimmune disease, characterized by autoantibody production and multi-organ damage. By a genome-wide association study (320 patients and 1,500 controls) and subsequent replication altogether involving a total of 3,300 Asian SLE patients from Hong Kong, Mainland China, and Thailand, as well as 4,200 ethnically and geographically matched controls, genetic variants in *ETS1* and *WDFY4* were found to be associated with SLE (*ETS1*: rs1128334, *P* = 2.33×10^−11^, OR = 1.29; *WDFY4*: rs7097397, *P* = 8.15×10^−12^, OR = 1.30). *ETS1* encodes for a transcription factor known to be involved in a wide range of immune functions, including Th17 cell development and terminal differentiation of B lymphocytes. SNP rs1128334 is located in the 3′-UTR of *ETS1*, and allelic expression analysis from peripheral blood mononuclear cells showed significantly lower expression level from the risk allele. WDFY4 is a conserved protein with unknown function, but is predominantly expressed in primary and secondary immune tissues, and rs7097397 in *WDFY4* changes an arginine residue to glutamine (R1816Q) in this protein. Our study also confirmed association of the *HLA* locus, *STAT4*, *TNFSF4*, *BLK*, *BANK1*, *IRF5*, and *TNFAIP3* with SLE in Asians. These new genetic findings may help us to gain a better understanding of the disease and the functions of the genes involved.

## Author Summary

In this study, we first conducted a genome-wide association study in a Hong Kong Chinese population, followed by replication in three other cohorts from Mainland China and a cohort from Thailand, which totaled 3,300 Asian patients and 4,200 ethnically and geographically matched controls. We identified novel variants in *ETS1* and *WDFY4* associated with SLE with genome-wide significance and confirmed the association of *HLA* locus, *STAT4*, *BLK*, *IRF5*, *BANK1*, *TNFSF*, and *IRF5* with the disease. *ETS1* encodes a critical transcription factor involved in Th17 and B cell development. Allelic expression study showed a significantly lower expression of *ETS1* from the risk allele, which provided functional support to the genetic findings. WDFY4 is a huge protein with unknown function but is predominantly expressed in primary and secondary immune tissues, and a nonsynonymous SNP in this gene was found to be highly associated with SLE susceptibility. Our findings shed new light on the function of these genes as well as the mechanism of this devastating disease.

## Introduction

Systemic lupus erythematosus (SLE) is a prototype autoimmune disease characterized by auto-antibody production and multi-organ damage. Genetic factors are known to play an important role in the disease, with the monozygotic twin concordance rate between 20–59, and the risk for siblings of affected individuals 30 times higher than that for the general population [1]–[3]. There are also population differences for the disease both in terms of genetic susceptibility and disease manifestations. African Americans, Hispanics and Asians all have higher disease prevalence than Caucasians; with Asians known to have more lupus nephritis than patients of European ancestry [4],[5].

Genome-Wide Association studies (GWAS) have dramatically changed the landscape of SLE genetics, with a pace of discovery the field has never seen before. In less than two years time, *STAT4* [6], *ITGAM* [7]–[9], *BLK* [8], *PXK* and *KIAA1542* [7], *BANK1* [10] and *TNFAIP3* [11],[12] and several other genes have been identified as associated with SLE [13]–[16]. More susceptibility loci were reported recently in two other GWAS on this disease [17],[18].

Despite varied disease prevalence and severity across different populations, it is noteworthy that most previous studies were conducted on patients of European ancestry with under-representation of other ethnicities. We previously examined some of the GWAS findings from populations of European origin [19]. In addition to confirming the association of *STAT4* and *BLK* with SLE in our population, our data indicated differences between the Asian and Caucasian populations. For example, our study did not detect any significant disease association for *PXK*, a result that was later confirmed by an independent study on a Korean population [20]. Data from our study indicated that, although the risk alleles in *ITGAM* are rare in Asians (<2%), they are risk factors in our populations and are closely related to lupus nephritis in particular [21].

In this study, we first genotyped 320 SLE patients collected in Hong Kong by the Illumina 610-Quad Beadchip and analyzed the data against 1500 control individuals genotyped on the same platform. Selected SNPs were then replicated in four independent sample collections from Hong Kong, Shanghai and Anhui, (China), as well as Bangkok (Thailand). Genetic variants in and around two genes, *ETS1* and *WDFY4*, were identified as associated with SLE with genome-wide significance. Functional characterization of the risk alleles also supported potential roles of these genetic variants in disease pathogenesis.

## Results

### The association of *HLA*, *STAT4*, *BLK*, *BANK1*, *IRF5*, *TNFAIP3* with SLE

**Figure 1:**
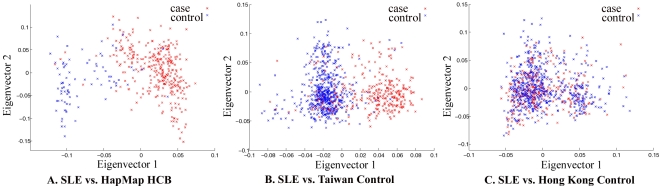
Principal component analysis of Chinese samples collected in Hong Kong, Taiwan, and Beijing. The red dots denote SLE patient samples collected in Hong Kong used in our GWAS study and the blue dots are unaffected samples from Beijing (A), Taiwan (B), and Hong Kong (C). The data on Taiwan samples was received from deCODE Genetics; the data on Han Chinese in Beijing is available from HapMap. The data on Hong Kong controls were from other studies in the University of Hong Kong that were genotyped by the same platform. Repeat of Eigenstrat analysis for 20 times using randomly chosen 100,000 SNPs each time from all the available SNPs produced similar results.

The whole-genome genotyping data was thoroughly examined by quality control measures and by population substructure analysis. Analysis of principal component using Eigenstrat [22] did reveal that the samples collected in Hong Kong clustered together, suggesting that confounding population substructure or admixture is not a major concern if Hong Kong controls were used in association analyses. It did indicate, however, that samples collected in Taiwan (obtained from deCODE Genetics) and Han Chinese in Beijing (HCB, available from HapMap) cluster very differently from Hong Kong samples, suggesting population substructure among Chinese living in different geographical regions and potential pitfalls in association studies when cases and controls are not well-matched (Figure 1).

**Figure 2:**
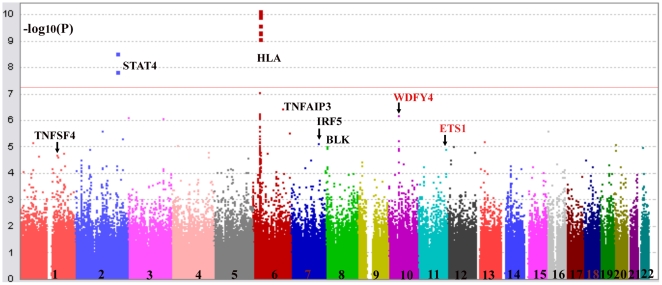
Confirmation of association of *HLA* locus, and *TNFSF4*, *STAT4*, *TNFAIP3*, *IRF5*, *BLK* with SLE. Shown are association results comparing SLE patients with controls collected in Hong Kong analyzed by Plink (−log_10_(*P*-value) of SNPs). The best SNP in Chromosome 11 is around *ETS1* gene (rs6590330), which is in high LD with rs1128334. And the best SNP in Chromosome 10 is in *WDFY4* (rs877819).

Genome-wide association analysis confirmed significant association of some established susceptibility genes in our population, including SNPs in the *HLA* locus, *STAT4*, *TNFSF4*, *BANK1*, *TNFAIP3*, *IRF5* and *BLK* (Table 1 and Figure 2). Similar to the Caucasian findings [12],[23], the risk allele for rs2230926 in *TNFAIP3* is low in frequency but with a relatively large effect size in disease association. SNP rs9271366 located between *HLA-DRB1* and *HLA-DQA1* is the most significantly associated SNP in the whole genome in our study. Although the *HLA* locus has been consistently shown to be the locus conferring the largest effect size with SLE association, there is little overlap between previous GWAS findings from populations of European ancestry and our results. The most significant SNPs in the Caucasian data [7],[8] are either monomorphic in our population or are not associated with disease susceptibility based on our GWAS result, something worth further pursuit in future studies. Association of *STAT4*, *BLK*, *IRF5*, *BANK1* and *TNFSF4* with the disease has been reported in our population previously [19],[21],[24],[25].

**Table 1:** Confirmation of susceptibility genes in the Hong Kong Chinese population by GWAS.

Genes	SNP	CHR	POSITION	A1	F_A	F_U	A2	P	OR
*TNFSF4*	rs1234315	1	171445086	T	0.5394	0.4678	C	1.04E-03	1.33
	rs704840	1	171492818	G	0.4905	0.4204	T	1.21E-03	1.33
*STAT4*	rs7574865	2	191672878	T	0.4589	0.3356	G	4.22E-09	1.68
	rs10168266	2	191644049	T	0.4543	0.3369	C	2.17E-08	1.64
*BANK1*	rs4522865	4	102934911	G	0.3801	0.4577	A	3.56E-04	0.73
	rs10516487	4	102970099	T	0.1392	0.1900	C	2.82E-03	0.69
*HLA-DRB1/HLA-DQA1*	rs9271366	6	32694832	G	0.2231	0.1283	A	7.67E-10	1.95
*HLA-DQB1/HLA-DQA2*	rs9275328	6	32774800	T	0.1656	0.2594	C	5.73E-07	0.57
*TNFAIP3*	rs2230926	6	138237759	G	0.0599	0.0225	T	3.42E-07	2.77
	rs3757173	6	138231847	C	0.0678	0.0356	T	2.08E-04	1.97
*IRF5*	rs729302	7	128356196	C	0.2539	0.3446	A	1.01E-05	0.65
	rs4728142	7	128361203	A	0.1703	0.1201	G	6.13E-04	1.50
*BLK*	rs2736340	8	11381382	C	0.2098	0.2974	T	8.56E-06	0.63
	rs2254546	8	11381089	A	0.1877	0.2720	G	1.03E-05	0.62

A1: minor allele; F_A: minor allele frequency in cases; F_U: minor allele frequency in controls.

**Figure 3:**
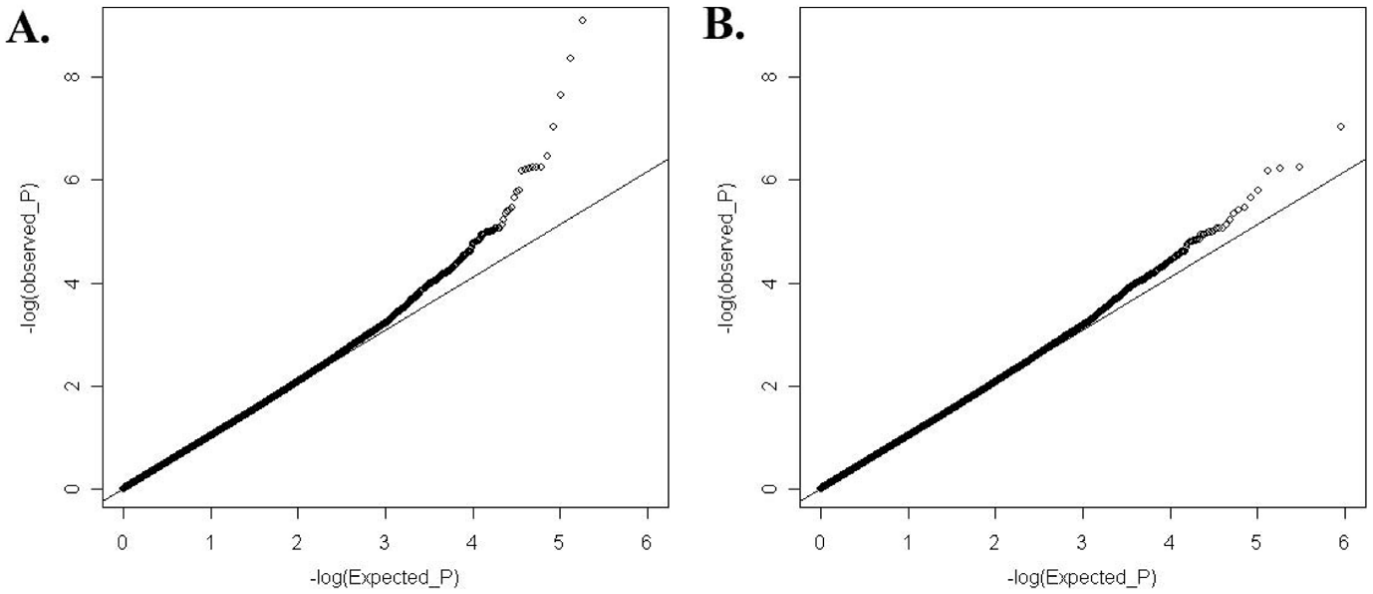
Quantile-Quantile plot of expected (x-axis) and observed (y-axis) −log_10_(*P* value) distribution in our GWAS analysis. (A) Considering all the available SNPs. (B) SNPs in and around *HLA locus*, and *TNFSF4*, *STAT4*, *TNFAIP3*, *IRF5*, *BLK*, as well as *BANK1* were excluded from analysis.

To answer the question on whether there are still other genetic variants contributing to disease susceptibility, we reexamined the Q-Q plot comparing expected and observed *P* values by removing all the SNPs in the known susceptibility loci mentioned above. After removal of these SNPs, we still observed an excess of association signal (Figure 3), suggesting involvement of additional susceptibility loci for this disease. Since our GWAS involved a limited number of patients, and is therefore prone to false positive and false negative findings, we selected SNPs for replication based on both their significance in GWAS results as well as the function and expression pattern of the nearby genes. Selected SNPs were replicated first using Sequenom genotyping on limited number of additional samples (360 cases and 360 controls), and variants with significant association in the Sequenom data were then examined by TaqMan genotyping on a much expanded sample collection from four independent cohorts. SNPs in and around two genes, v-ets erythroblastosis virus E26 oncogene homolog 1 (avian) (*ETS1*) and the WDFY family member 4 (*WDFY4*) regions were chosen based both on initial GWAS data as well as their known function (in the case of *ETS1*) and expression pattern (in the case of *WDFY4*). Table 2 displays the SNPs in these two loci that showed disease association with a *P* value<0.01 from our GWAS data. Initial replication by Sequenom showed consistent results with the GWAS trend for these two genes and they were further tested in the remaining samples.

**Table 2:** SNPs in and around *ETS1* and *WDFY4* that showed significant association with SLE in the GWAS data.

	SNP	CHR	POSITION	A1	F_A	F_U	A2	P	OR
*ETS1*	rs6590330	11	127816269	A	0.4401	0.3466	G	8.92E-06	1.48
	rs7932088	11	127822378	T	0.3312	0.3893	G	6.24E-03	0.78
	rs10893872	11	127830763	T	0.5142	0.4299	C	1.04E-04	1.40
	rs4937333	11	127835730	T	0.5110	0.4279	C	1.28E-04	1.40
	rs12223943	11	127900838	T	0.1009	0.1409	G	7.25E-03	0.68
*WDFY4*	rs10508908	10	49643864	A	0.5047	0.4356	G	1.47E-03	1.32
	rs11595065	10	49647888	G	0.5142	0.4379	A	4.54E-04	1.36
	rs6537575	10	49649705	T	0.4432	0.3819	C	4.08E-03	1.29
	rs2947344	10	49654696	T	0.5284	0.4574	C	1.14E-03	1.33
	rs2663031	10	49657309	A	0.5284	0.4574	G	1.14E-03	1.33
	rs2448541	10	49684822	G	0.4416	0.3532	A	2.76E-05	1.45
	rs2448539	10	49685217	A	0.2310	0.1768	C	1.49E-03	1.40
	rs10857650	10	49700029	G	0.3644	0.2735	A	4.63E-06	1.52
	rs877819	10	49712957	A	0.2618	0.1758	G	5.66E-07	1.66
	rs7922169	10	49715462	T	0.3549	0.2678	G	9.83E-06	1.50
	rs11101535	10	49734437	T	0.4669	0.3960	C	9.73E-04	1.34
	rs2663049	10	49736767	C	0.3517	0.2654	T	1.12E-05	1.50
	rs2663052	10	49739401	C	0.3517	0.2651	T	1.03E-05	1.50
	rs2620881	10	49753117	A	0.3339	0.2609	G	2.42E-04	1.42
	rs2671692	10	49767825	A	0.3281	0.2594	G	4.14E-04	1.39
	rs2943244	10	49770895	C	0.2476	0.1798	A	8.10E-05	1.50
	rs2663041	10	49777543	C	0.3502	0.2722	T	7.96E-05	1.44
	rs11101558	10	49783035	A	0.3312	0.2631	G	4.85E-04	1.39
	rs1913517	10	49789060	A	0.3328	0.2617	G	2.70E-04	1.41
	rs7094610	10	49792187	C	0.3344	0.2780	A	4.44E-03	1.30

### Association of *ETS1* with SLE

**Figure 4:**
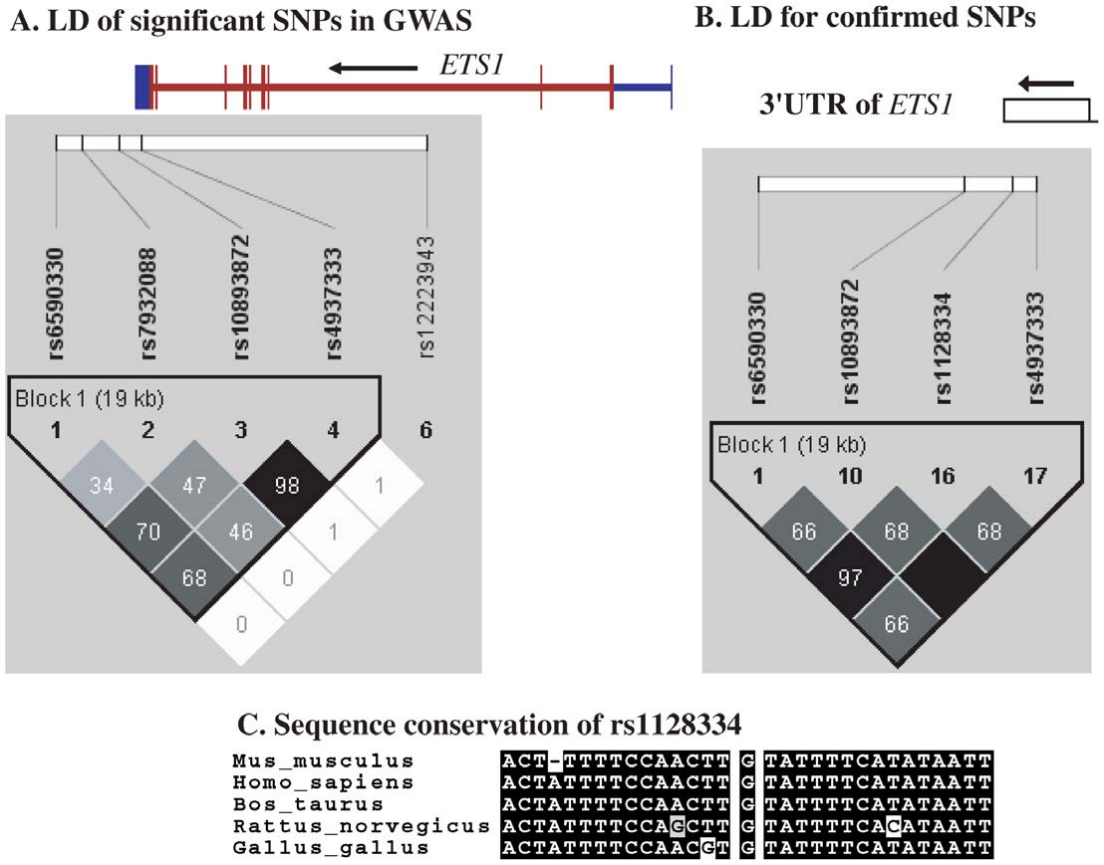
Linkage disequilibrium and sequence conservation of SNPs in *ETS1*. Shown are LD for significant SNPs in and around *ETS1* detected by GWAS (A), and replicated SNPs in the 3′-UTR and downstream of the gene (B), and sequence conservation for sequences around SNP rs1128334 among different species (C).

Making use of the remaining samples from Hong Kong not included in GWAS, and sample collections from Shanghai and Anhui, China, and Bangkok, Thailand, we went on to replicate the whole-genome findings on these two loci. SNPs in *ETS1* listed in Table 2, rs12223943, rs7932088 and rs10893872, were examined in the expanded samples. SNPs rs10893872 and rs4937333 have absolute LD with each other, so only rs10893872 was chosen for replication. SNP rs1128334 was chosen in the place of rs6590330 for replication due to its high LD with rs6590330 (r^2^ = 0.97, Figure 4) and its relative position to the gene (3′-UTR) and predicted effect on microRNA binding. For SNPs rs12223943 and rs7932088, the association seen from whole-genome data was inconclusive (data not shown) in the replication stage and were not further pursued. We genotyped rs10893872 and rs1128334 in all the samples from the four cohorts and both were found to be highly associated with SLE (Table 3).

**Table 3:** SNPs showed significant association with SLE in a joint analysis of four independent Asian cohorts.

SNP	Chr	Nearby Gene	Location relative to gene	MINOR ALLELE FREQUENCIES (MAF, %)								JOINT *P* VALUE	OR	95% CI OF OR
				Hong Kong		Shanghai		Bangkok		Hefei, China				
				Case N = 1073	Cont N = 1742	Case N = 920	Cont N = 1053	Case N = 314	Cont N = 519	Case N = 951	Cont N = 860			
rs1128334	11	*ETS1*	3′-UTR	40.6	35.1	38.7	34.5	35.1	31.5	39.8	29.6	2.3E-11	1.29	1.20–1.39
rs10893872	11	*ETS1*	downstream	47.3	42.8	45.6	42.1	49.1	42.3	44.0	38.1	1.8E-7	1.21	1.13–1.31
rs7097397	10	*WDFY4*	Coding, R1816Q	35.3	27.5	37.2	34.3	41.9	33.8	39.2	33.6	8.15E-12	1.30	1.21–1.40
rs877819	10	*WDFY4*	intron	23.4	16.4	17.3	18.6	28.3	22.2	20.6	17.3	5.57E-7	1.26	1.15–1.37

Independence test by logistic regression by Plink pointed to a major contribution from rs1128334. And indeed, the sequence around rs1128334 has high sequence conservation among different species (Figure 4). Haplotype analysis indicated that the TA haplotype formed by the two SNPs (rs10893872 and rs1128334) is the major risk haplotype, whilst the CG haplotype is the major protective haplotype (Table 4) with other haplotypes having low allele frequencies. Subphenotype analysis was also performed for these SNPs, and both SNPs were found to have larger effect sizes for patients with lupus nephritis in all four cohorts, although no statistical significance was reached in any cases. Analysis of other subphenotypes showed insignificant, or inconsistent results among different cohorts.

**Table 4:** *ETS1* Haplotype analysis on SLE association.

rs10893872-rs1128334		F_A (%)	F_U(%)	*P* - Value
TA haplotype	Hong Kong	40.35	33.2	5.89E-06
	Anhui	36.99	28.51	1.25E-04
	Bangkok	35.58	31.02	0.06
	Shanghai	37.93	32.93	0.0023
CG haplotype	Hong Kong	51.86	56.63	0.0035
	Anhui	53.71	62.31	0.00022
	Bangkok	50.76	57.28	0.0113
	Shanghai	53.35	56.06	0.11

### Allelic expression of *ETS1* in PBMC

**Figure 5:**
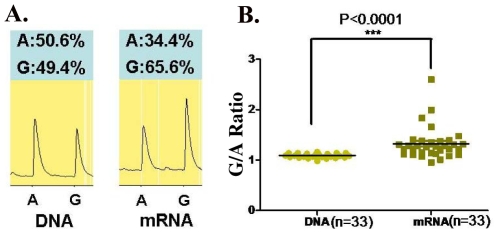
Allelic expression of *ETS1* on SNP rs1128334 in PBMC of healthy individuals. PBMC cDNA processed from 33 healthy individuals heterozygous on rs1128334 were used for allelic expression detection of *ETS1* by pyrosequencing. (A) A case example of detection on the “A” allele and the “G” allele from both DNA and cDNA samples from the same individual. (B) The ratio of G/A allelic detection for both DNA and cDNA samples. The median G/A ratio for DNA is 1.09 (95% CI: 1.08–1.11) and the median G/A ratio for cDNA expression is 1.32 (95% CI: 1.21–1.43), *P*<0.0001 by paired student's *t* test.

Since rs1128334 is located at the 3′-UTR region of the gene, it is predicted that it may have an effect on the expression level of *ETS1*. Therefore, we examined allelic expression of *ETS1* gene for the two alleles of rs1128334, “A” and “G”, from healthy individuals heterozygous for this SNP (N = 33). This assay assesses directly whether the two alleles of the SNP correlate with different steady state mRNA levels. Pyrosequencing results from PBMC of healthy individuals heterozygous on the SNP showed a significantly higher expression from the “G” allele than from the risk “A” allele, with a *P* value<0.0001 (Figure 5).

### Association of *WDFY4* with SLE

Two of the SNPs in *WDFY4* that showed the most significant association with the disease in our GWAS, SNPs rs10857650 and rs877819, were selected for further replication. In addition, three nonsynonymous SNPs in this gene not genotype by the Illumina 610-Quad Beadchip, rs2170132 (Ser1528Pro), rs7097397 (Arg1816Gln) and rs2292584 (Pro3118Leu), were also selected to test for disease association, aiming at identifying functional variants in this gene. SNPs rs2170132 and rs2292584 did not show significant difference between the cases and the controls in the Hong Kong cohort (Table 5) and Thai samples (data not shown) and were not further tested in other cohorts. Genotyping results on rs10857650 using TaqMan showed significant discordance with results from the Illumina Beadchip, and was thus removed from further analysis.

**Table 5:** Replication of disease association for *WDFY4* SNPs in the Hong Kong samples.

SNP	Position to the gene	BP	A1	F_A	F_U	A2	CHISQ	P	OR
Rs2170132	Ser1528Pro	50013402	T	0.1679	0.1617	C	0.2793	0.5971	1.046
rs7097397	Arg1816Gln	50025396	G	0.3534	0.2753	A	2.88E+01	7.88E-08	1.438
rs877819	intron	50042951	A	0.2339	0.1639	G	3.12E+01	2.33E-08	1.557
Rs2292584	Pro3118Leu	50816415	T	0.3679	0.3416	C	3.075	0.0795	1.122

**Figure 6:**
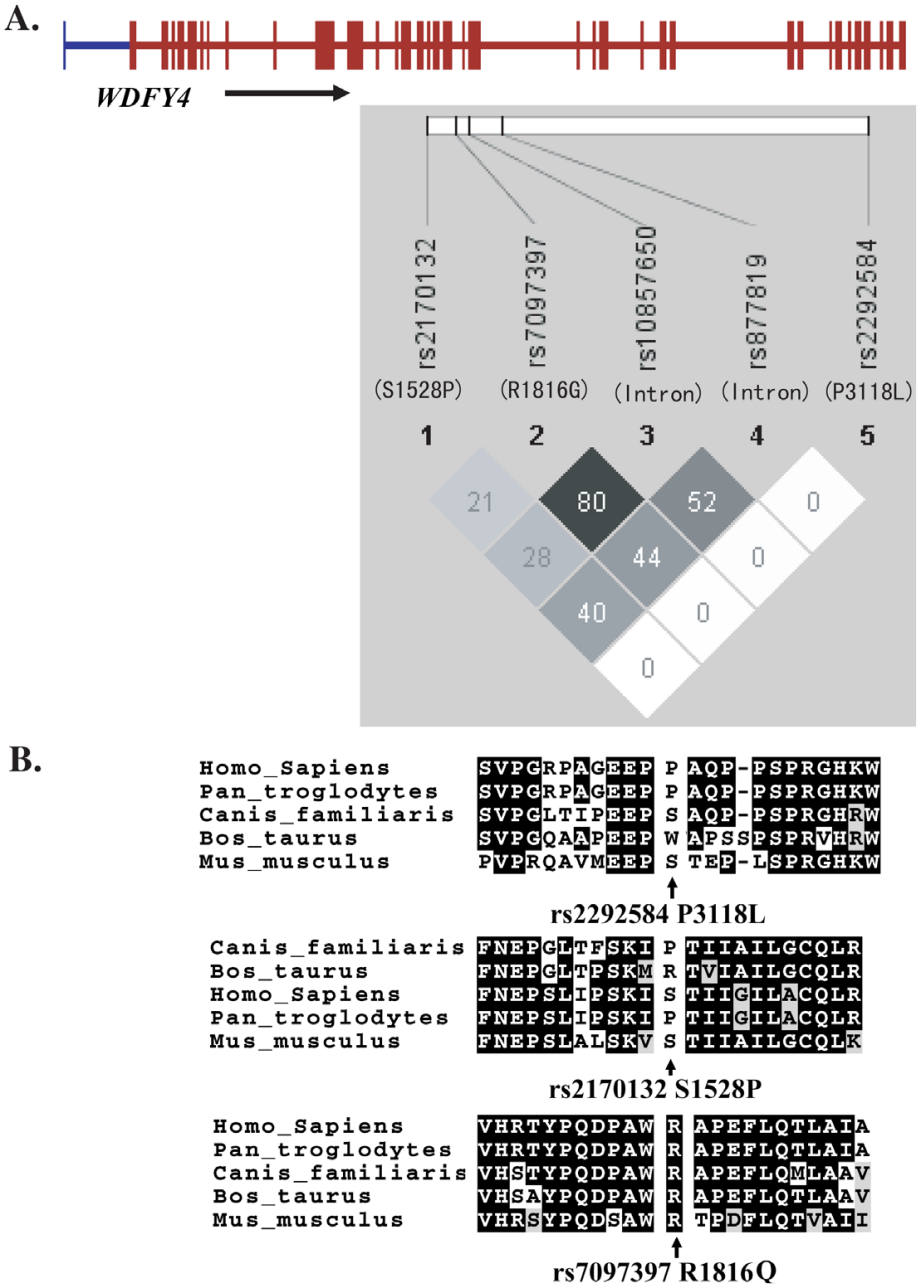
LD among *WDFY4* SNPs examined in this study (A) and sequence conservation of the three nonsynonymous variations among various species (B).

SNP rs7097397 and rs877819 were confirmed to have significant association with the disease (Table 3). Conditional logistic regression test indicate that the nonsynonymous SNP coded by rs7097397 is probably the functional variant, with *P* = 1.01×10^−5^ when controlling the effect of rs877819. Independent contribution from rs877819 is questionable, with a *P* value of 0.088 considering the effect of rs7097397 in the same test. The two SNPs have intermediate LD (r^2^ = 0.44, Figure 6A). The genetic result is consistent with the fact that the arginine residue at 1816 in WDFY4 protein is well conserved among orthologs in different mammals (Figure 6B). Preliminary analysis on subphenotype stratification suggests that this nonsynonymous change may be more closely involved in male and early onset patients in a case only analysis (onset age 12 and below vs. above, OR = 1.96, *P* = 0.0021; male vs. female: OR = 1.52, *P* = 0.023).

Association of the SNPs in the two genes with disease risk was also corrected by logistic regression using age and sex as covariates, and the associations found in this study all remain highly significant after all the corrections.

## Discussion

Several GWAS on SLE have been conducted on populations of European ancestry [7],[8], but populations of Asian or African ancestry were seriously underrepresented. Only during the process of submission of our current work, a GWAS study on Chinese populations was reported [18]. Considering the population differences in both disease prevalence and clinical manifestations, GWAS on non-Caucasian populations may have novel findings and help to elucidate the differences between populations.

An interesting analysis result from our GWAS data is the difference between Hong Kong samples and samples collected in Taiwan and Beijing, shown by principal component analysis (Figure 1). It suggests population substructure for Chinese living in different regions, which may cause spurious findings in association studies when cases and controls are not well matched. With most of the genetic variants of relatively larger effect sizes already being identified, GWAS becomes more susceptible to effects from mismatches between cases and controls in dealing with SNPs of smaller effect sizes. Our analysis echoed two very recent reports delineating population substructures in Chinese populations living in different geographical regions [26],[27].

**Figure 7:**
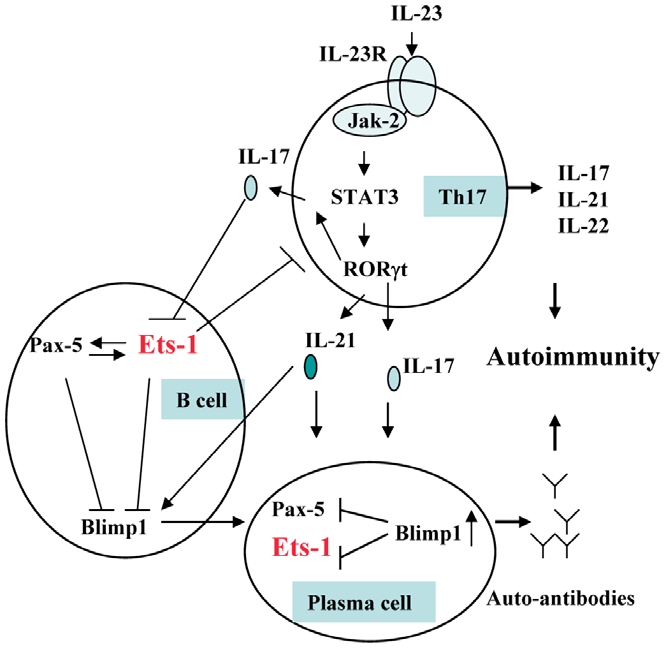
Involvement of *ETS1* in Th17 cell and B lymphocyte development and autoimmunity.

Ets-1 is a member of the ETS family of transcription factors that share a unique Ets DNA binding domain. They control a wide variety of cellular processes including cell proliferation and differentiation [28]. Ets-1-deficient mice develop lupus-like disease characterized by high titers of IgM and IgG autoantibodies, immune complex-mediated glomerulonephritis, and local activation of complement [29]. Ets-1 is also involved in many cellular abnormalities that are known to participate in SLE pathogenesis as illustrated in Figure 7.

Ets-1 is a negative regulator of terminal differentiation of B cells and plays critical roles in maintaining B cell identity [30]–[32]. Ets-1-deficient B cells were present in normal numbers but have a large proportion of IgM plasma cells [33]. Ets-1 blocks the function of B-lymphocyte-induced maturation protein 1 (Blimp-1), an essential transcription factor for plasma cells [34]. The number and frequency of plasma cells were known to correlate with disease activity and the titer of anti-dsDNA antibodies in SLE [35],[36].

Ets-1 is also a negative regulator of Th17 cell differentiation, and naïve CD4^+^ T cells deficient in Ets-1 undergo greatly enhanced differentiation into Th17 cells when cultured *in vitro* under Th17-skewing conditions [37]. Th17 cells with specificity for self-antigens are known to be highly pathogenic and lead to the development of inflammation and severe autoimmunity [38]. Higher plasma IL-17, IL-23 and higher number of Th17 cells in SLE patients were reported and correlated positively with SLE disease index (SLEDAI) [39]–[41]. IL-17 and IL-21 produced by Th17 cells may also induce B cell terminal differentiation [42]–[44].

In this study, we found that in the PBMC of healthy individuals, expression of *ETS1* from the risk “A” allele is reduced comparing to that from the “G” allele. The expression level of *ETS1* may be tightly regulated. It was shown that resting T cells express high levels of *ETS1* mRNA and protein, which decreased to very low levels upon T cell activation [45]. Lower expression of *ETS1* for the risk allele carriers may play a role in disease pathogenesis through increased differentiation and activity of both plasma cells and Th17 cells.

It is likely that the association SNPs identified in this study may affect the response of *ETS1* gene to other upstream signals. SNP rs1128334 locates in the 3′-UTR of *ETS1*, and rs10893872 is in absolute LD with rs4937333, another SNP that is also located in the 3′-UTR of the gene and both SNPs are on putative microRNA (miRNA) binding sites. In a recent study by Du et al, the expression level of a microRNA, miR-326, was found to be related to disease severity in patients with multiple sclerosis and mice with experimental autoimmune encephalomyelitis. *ETS1* was shown to be the major target of miR-326, through downregulation of which miR-326 promoted the generation of Th17 cells both *in vitro* and *in vivo* [46]. Another microRNA, miRNA-146a, was also found to be involved in SLE pathogenesis [47].

WDFY family member 4 (*WDFY4*, NCBI GeneID: 57705) codes for a huge protein (3184 amino acid residues) with unknown function. Its closest paralog is WD repeat and FYVE domain containing 3 (*WDFY3*, NCBI GeneID: 23001). Similar to WDFY3, WDFY4 does contain WD40 domains and a BEACH (Beige and chediak-kigashi) domain, although FYVE zinc finger domain is truncated. Its sequence is well conserved among various species. For example, there is an 84% sequence similarity between the human protein and its orthologs from *Bos Taurus* and *Canis lupus familiaris*. And there is a 42% identity between the human protein and protein XP_701288.3 in *Danio Rerio* (zebra fish). Although protein XP_701288.3 is annotated as WDFY3 in NCBI, it has a higher sequence similarity with human WDFY4 than with WDFY3.

WD40 domain is found in a number of eukaryotic proteins that cover a wide variety of functions including adaptor/regulatory modules in signal transduction, pre-mRNA processing and cytoskeleton assembly, while the BEACH domains are implicated in membrane trafficking. While very little is known about the potential function of this well conserved protein, an interesting phenomenon is that the gene is predominantly expressed in immune tissues such as lymph node, spleen, thymus and tonsil (UniGene Hs.287379, http://www.ncbi.nlm.nih.gov/UniGene/ESTProfileViewer.cgi?uglist=Hs.287379), unlike *WDFY3*, which is expressed in a wide variety of tissues and organs.

After submission of our work, Han et al reported their GWAS work on SLE on Chinese populations [18] and reported genome-wide significant association signals on *ETS1* (rs6590330, downstream of the gene) and *WDFY4* (rs1913517, in the intron of both *WDFY4* and *LRRC18*, leucine rich repeat containing 18). Independent identification of these two genes in SLE susceptibility underlined the validity of the findings. Identifying susceptibility genes provided a good start in our effort to elucidate the disease mechanisms and the functions of the genes involved, although many questions remain to be answered. Characterization of the functions of the genes in autoimmunity may eventually help us to translate genetic findings into clinical and therapeutic applications.

## Materials and Methods

### Ethics statement

This study was conducted according to the principles expressed in the Declaration of Helsinki. The Hong Kong study was approved by the Institutional Review Board of the University of Hong Kong and Hospital Authority, Hong Kong West Cluster, New Territory West Cluster, and Hong Kong East Cluster. The study on Shanghai, Anhui and Thai samples was approved by the Institutional Review Board of Renji Hospital, Research Ethics Committee of Anhui Medical University and the Ethics Committee of the Faculty of Medicine, Chulalongkorn University, respectively. All patients provided written informed consent for the collection of samples and subsequent analysis.

### Subjects

1073 SLE samples collected in Hong Kong were from four hospitals in Hong Kong Island and the New Territories: Queen Mary Hospital, Tuen Mun Hospital, Queen Elizabeth Hospital and Pamela Youde Nethersole Eastern Hospital. The patients were all of self-reported Chinese ethnicity living in Hong Kong. The average onset age was 28 years old and the ratio of female to male patients was 9∶1. About half of the patients had renal involvement, and about 70% tested positive for anti-dsDNA antibodies. The SLE samples collected in Shanghai were patients attending Renji Hospital of Jiaotong University Medical School, a tertiary referral hospital covering Shanghai and the surrounding areas. There is an 8∶1 female to male patient ratio, and about 52% of patients have lupus nephritis. 951 SLE patients collected in Anhui were all self-reported Chinese ethnicity living in Anhui province, central China. They were recruited from the Departments of Rheumatology at Anhui Provincial Hospital and the First Affiliated Hospital of Anhui Medical University, both located in Hefei, Anhui province, about 450 km from Shanghai. The average onset age was 31 years old and the ratio of female to male patients was 17∶1. 314 Thai patients with SLE (female∶male ratio = 14∶1) attending King Chulalongkorn Memorial Hospital, a tertiary referral center in Bangkok were also recruited in this study. Medical records were reviewed to confirm that all subjects met the revised criteria of the American College of Rheumatology for SLE diagnoses [48].

Controls used in the GWAS stage were from both healthy individuals and from other studies conducted in the same institution genotyped with the same platform. For the replication stage, Hong Kong controls were healthy blood donors kindly contributed by the Hong Kong Red Cross and were all of self-reported Chinese ethnicity living in Hong Kong. Controls from Shanghai and Anhui were selected from a pool of healthy blood donors recruited from Renji Hospital (Shanghai) and Hefei City (Anhui), respectively, with an effort to match for the age and sex of corresponding SLE patients. Thai controls were recruited from unrelated voluntary healthy donors from the same ethnic background and geographic area as the Thai SLE patients.

### Genome-wide association study

320 (27 males, 293 females) SLE patients were genotyped by Illumina 610-Quad Human Beadchip with a total number of SNPs reaching 620,901. 24 individuals were removed due to low call rate or hidden first degree relationship. A total number of 104,395 SNPs were also removed from the initial analysis on the ground of low genotyping call rate (<90%, 30,133 SNPs) and/or low minor allele frequency (MAF) (<0.005, 102,970 SNPs). 2,285 SNPs were also removed due to violation of Hardy-Weinberg equilibrium in controls (*P* values<0.0001). After quality control measures, 314 case (27 males, 287 females) and 1484 controls (840 males and 644 females) were analyzed on a total of 514,221 SNPs. The call rate for the remaining SNPs reached 0.999 with a genome-wide inflation factor of 1.03, which is an indication of good match between the cases and controls. To overcome any potential effect from the heterogeneity in the controls, three independent comparisons were initially conducted by separating the controls into three different subsets and only SNPs reaching significance in all subsets in association analysis were selected for further replication.

The SNPs were analyzed for association with the disease by means of comparison of the minor allele frequency in patients and controls (basic allelic test) as well as other tests using Plink [49]. Linkage disequilibrium (LD) patterns were analyzed and displayed by HaploView [50]. Association of the SNPs with disease risk was also corrected by logistic regression using age and sex as covariates. Average odds ratios (OR) and *P* values jointly analyzed from four sample collections were obtained by Cochran-Mantel-Haenszel (CMH) test of disease association conditional on SNP frequency differences among different populations. Test of independent contributions of a SNP controlling for the effect of other SNPs in the same locus was done by conditional logistic regression as well as haplotype analyses. Subphenotype stratification was performed by comparing cases with and without a given subphenotype.

### Genotyping in replication stage

SNPs rs1128334, rs10893872, rs7097397, rs10857650, and rs877819 were genotyped by TaqMan SNP genotyping method using assay-on-demand probes and primers (Applied Biosystems, Foster City, CA94404, USA). Some of the initial screening was also done using Sequenom MassARRAY iPLEX Gold system. Genotyping accuracy was confirmed by direct sequencing of PCR products for some randomly chosen samples. Genotyping concordance between Illumina Human 610-Quad Beadchip and TaqMan SNP genotyping method was also examined on selected samples and probes: rs877819 has a concordance rate of 99.64% (1 out of 277 differed by the two platforms); rs10893872 has a concordance rate of 99.16% (1 out of 119 samples differed). SNP rs1128334 and rs7097397 were examined by direct sequencing of selected samples and showed complete consistence between TaqMan and sequencing (50 samples each). The results of rs10857650 were discarded due to low concordance between the Illumina Beadchip data and the TaqMan results.

### Allele-specific transcription quantification

Thirty-three healthy individuals heterozygous for rs1128334 were chosen to assess the relative *ETS1* mRNA levels from the two alleles, “A” and “G”, by pyrosequencing [51],[52]. In the meantime, DNA detection ratio was used as a control for amplification efficiency. Briefly, total RNA was extracted from peripheral blood mononuclear cell (PBMC) from each individual. RNA samples were then treated with DNAase to eliminate genomic DNA contamination before being reverse-transcribed into cDNA using oligo-dT primer. cDNA was then amplified by PCR using transcript-specific primers, together with DNA from the same individuals. The cDNA and DNA PCR products were purified using the Qiaquick PCR purification kit, and then subjected to allele quantitative pyrosequencing. The sequencing primer was designed using Pyrosequencing Assay Design Software v.1.0. Reactions were performed on a Biotage PSQ96MA machine, and allele quantification was analyzed using PSQMA 2.1 software. The average G/A cDNA expression ratio of each individual was normalized by the G/A DNA ratio from the same sample. Paired student' *t* test was used to compare the normalized expression level from the “A” and “G” alleles.
